# From Nature to Nanomedicine: Green-Synthesized Metal Oxide Nanoparticles for Oral Cancer Drug Delivery

**DOI:** 10.3390/nano15231767

**Published:** 2025-11-25

**Authors:** Doaa S. R. Khafaga, Youssef Basem, Mariam Mohamed Ali, Rawan S. Elsharkawy, Ayda H. El-Gouhari, Shimaa S. Attia

**Affiliations:** 1 Department of Basic Medical Sciences, Health Sector, Galala University, New Galala City 43511, Suez, Egypt; 2 Medical and Pharmaceutical Industrial Biotechnology Department, College of Biotechnology, Misr University for Science and Technology, 6th of October City 12566, Giza, Egypt; 200025117@must.edu.eg; 3 Faculty of Biotechnology, Badr University in Cairo, Badr City 11829, Cairo, Egypt; mariammuhaammeddd23@gmail.com; 4 Faculty of Medicine, Galala University, New Galala City 43511, Suez, Egypt; rawan.sameh@gu.edu.eg (R.S.E.); ahe800165@gu.edu.eg (A.H.E.-G.); 5 Anatomy & Embryology Department, Faculty of Medicine, Ain-Shams University, Abaseya 11566, Cairo, Egypt; 6 Biomedical Sciences Department, College of Medicine, Gulf Medical University, Ajman 4184, United Arab Emirates

**Keywords:** nanomedicine, oral cancer, metal oxide nanoparticles, drug delivery, herbs, green synthesis

## Abstract

Oral cancer represents one of the most prevalent malignancies worldwide, characterized by high morbidity and mortality rates primarily due to late diagnosis, limited therapeutic efficacy, systemic toxicity, and recurrence following conventional treatments. Traditional chemotherapeutic drugs, while effective to a certain extent, often suffer from poor bioavailability, nonspecific targeting, and multidrug resistance, highlighting the importance of innovative therapeutic strategies. Nanomedicine has emerged as a promising alternative, providing site-specific delivery, enhanced drug stability, and improved therapeutic outcomes. Among various nanoparticles (NPs), metal oxide nanoparticles (MONPs), such as zinc oxide, titanium dioxide, and copper oxide, have demonstrated potent anticancer activity due to their high surface area, tunable physicochemical properties, and ability to generate reactive oxygen species (ROS). Recent progress in green synthesis approaches, employing plant extracts, microbes, and biopolymers as reducing and stabilizing agents, has further advanced the development of biocompatible and eco-friendly NPs. These green-synthesized NPs minimize toxic byproducts and allow their functionalization with herbal compounds and conventional drugs, offering synergistic effects against oral cancer. This review highlights the limitations of traditional treatments, examines the role of nanomedicine, and discusses the application of green-synthesized MONPs as drug delivery platforms for oral cancer management. It also addresses challenges such as standardization, scalability, safety concerns, and regulatory barriers, while outlining future perspectives that integrate green nanotechnology with precision medicine. Collectively, green nanomedicine offers a sustainable and innovative paradigm with the potential to revolutionize oral cancer therapy.

## 1. Introduction

Oral cancer, particularly oral squamous cell carcinoma (OSCC), represents one of the leading causes of cancer-related mortality worldwide and is ranked among the six most common malignancies globally [1]. Conventional treatment modalities, including surgery, chemotherapy, and radiotherapy, are often associated with severe side effects such as functional loss, disfigurement, and systemic toxicity, which significantly limit their therapeutic efficacy and adversely affect patients’ quality of life [2]. Recently, nanotechnology has emerged as a promising approach in medicine to improve therapeutic outcomes. Nanoscale systems for drug delivery offer the potential to selectively deliver therapeutic agents to tumor sites, thereby reducing systemic toxicity to healthy tissues while enhancing local drug accumulation within the tumor [3]. Among these systems, MONPs such as zinc oxide (ZnO), iron oxide (Fe_2_O_3_/Fe_3_O_4_), titanium dioxide (TiO_2_), copper oxide (CuO/Cu_2_O), and cerium oxide (CeO_2_) have attracted considerable attention. Their unique physicochemical properties, including the ability to generate ROS, facilitate efficient drug delivery and enable selective interactions within the tumor microenvironment, rendering them highly promising for oral cancer therapy [4,5]. Importantly, MONPs synthesized via eco-friendly routes, often referred to as “green synthesis,” offer additional advantages by enhancing therapeutic efficacy in a sustainable and environmentally responsible manner. This approach utilizes plant extracts or microbial metabolites as reducing and stabilizing agents, rather than hazardous chemical reagents. Green-synthesized NPs have demonstrated higher selectivity, reduced toxicity toward normal cells, and preserved anticancer activity compared with chemically synthesized counterparts [6].

Several studies have reported that green-synthesized MONPs exhibit significant anticancer potential, enhanced biocompatibility, and efficient tumor targeting. These NPs often benefit from the enhanced permeability and retention effect (EPR), which allows preferential accumulation within tumor tissues due to the leaky vasculature and poor lymphatic drainage characteristic of solid tumors [7]. Among MONPs, ZnONPs have garnered significant attention due to their low cost, relative safety, and ease of surface modification, which facilitates precision targeting. Their intrinsic ability to generate ROS enables the selective induction of apoptosis in cancer cells. Moreover, ZnONPs have been investigated as carriers for conventional chemotherapeutics such as doxorubicin, further improving the drug’s intracellular delivery to oral tumor tissues [5].

Nevertheless, specific challenges remain to be addressed before clinical translation. These include ensuring long-term biocompatibility, evaluating degradation products, and confirming the safety of repeated or chronic administration, especially within the complex and sensitive oral cavity microenvironment [7]. The current review aims to provide a comprehensive overview of recent advances in the green synthesis of MONPs and their application as targeted drug delivery systems for various anticancer drugs, highlighting their expected to be effective in oral cancer. Particular emphasis will be placed on their therapeutic and environmental advantages, as well as the challenges that must be overcome to achieve clinical translation, including enhanced targeting efficiency, minimization of systemic toxicity, and long-term biosafety evaluation. Furthermore, combining nanomaterials with herbal compounds can overcome the limitations of herbs used in oral cancer therapy and synergistically enhance the anticancer effect when co-administered with conventional drugs.

## 2. Methodology

**Figure 1:**
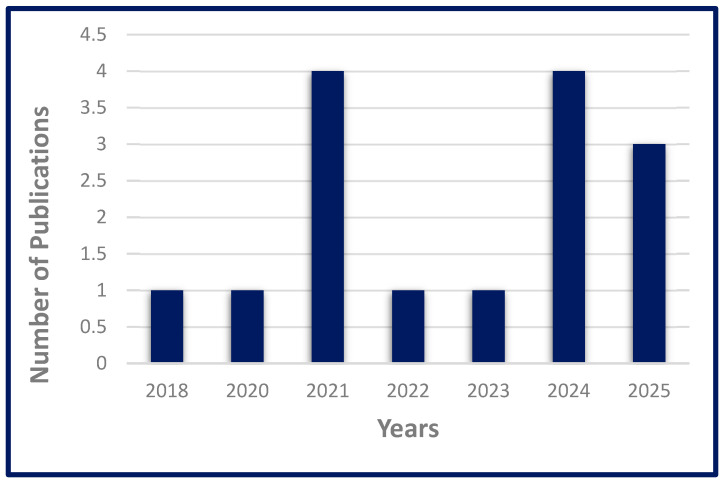
Number of publications through the Scopus database using a combination of keywords (2018–2025).

Comprehensive searches were conducted through PubMed, Scopus, Web of Science, ScienceDirect, and Google Scholar, utilizing combinations of relevant keywords, including “cancer”, “green synthesis”, “metal oxide”, and “nanomedicine”, covering the period from 2018 to 2025, as illustrated in Figure 1. Eligible studies included experimental research, systematic reviews, and narrative reviews that directly addressed the application of green-synthesized MONPs in cancer therapy. Non-English articles and studies not related to cancer or nanomedicine were excluded.

## 3. Limitations of Traditional Drugs for Oral Cancer Treatment

Drugs continue to serve as a fundamental component in treating numerous diseases, with their therapeutic efficacy dependent on their pharmacological category, mechanism of action, and clinical use. At the same time, each agent’s risk–benefit profile must be carefully considered, as adverse effects can limit their use or necessitate alternative strategies, as summarized in Table 1.

## 4. Nanomedicine Mechanism of Action in Oral Cancer

Nanomaterials have external dimensions 1–100 nm, according to number size distribution [24]. It has been demonstrated that nanotechnology can mitigate many of the drawbacks of conventional chemotherapy and serves as valuable tool for enhancing the overall effectiveness of cancer treatment. Drug molecules are typically encapsulated, adsorbed, dissolved, trapped, or affixed to nanomatrices. NPs provide tumor-selective accumulation of their payloads and can improve the bioavailability of drugs with low water solubility. Most significantly, chemotherapeutic drugs preferentially accumulate in tumors, thereby improving therapeutic efficacy and lowering systemic toxicity. The effects of EPR are the central tenet of using NPs for cancer treatment. For passive targeting and long-term retention of NPs at the tumor site, EPR effects rely on high vascular permeability and decreased lymphatic outflow in solid tumors. Consequently, nanomedicines minimize the dose-dependent toxicity of chemotherapeutic drugs while significantly enhancing therapeutic outcomes. Several nanomedicines have been approved based on EPR effects, which have evolved to form the foundation of NP-based cancer therapy over time [25]. 

There are many methods of drug loading and release; for example, encapsulation, as numerous OSCC studies load photosensitizers, doxorubicin, paclitaxel, or cisplatin into liposomes or polylactic-co-glycolic acid (PLGA) NPs to preserve the medicine and increase local tumor concentrations. Compared to free drugs, OSCC models both in vitro and in vivo exhibit better cytotoxicity and less systemic exposure [26]. Surface adsorption of photosensitizers or chemotherapeutics onto surfaces for photothermal therapy (PTT) or photodynamic therapy (PDT) combination methods in OSCC is frequently achieved using gold nanorods or carbon-based nanomaterials [27]. Covalent conjugation with cleavable linkers is another common strategy. Acid-labile or disulfide linkers are commonly used in OSCC NP research to take advantage of intracellular glutathione for intracellular release or tumor/endosomal acidity. Several OSCC-focused studies demonstrate selective payload release in tumor cells via redox-cleavable bonds or pH-sensitive polyethylene glycol (PEG) detachment. To improve local dosage while reducing systemic toxicity, mucoadhesive NPs and local gel or spray formulations that lengthen residence duration on the oral mucosa are frequently researched for local OSCC treatment [26].

Nanoplatforms enhance drug concentration and reduce harm to healthy tissues by preferentially accumulating in the tumor microenvironment (TME). After tumor access and accumulation, the nanomedicine penetrates through oral tissues and TME by many mechanisms, like mucoadhesion and epithelial permeation, though mucoadhesive carriers such as chitosan enhance the adhesion to oral mucosa and paracellular transport, by which tissue penetration and residence time increase. On the other hand, there is another type of mechanism for penetration through a self-adhesive transmucosal system, utilizing mucoadhesive patches, which block tissue mobility and salivary flow, and are particularly useful for direct lesion delivery. Mucoadhesion ensures the continuous presence of medication for long-term administration by relying on physicochemical interactions between sticky polymers and the mucus layer [28]. Despite variations in the oral cavity, these methods also preserve the ideal medication concentration. Strong bio-adhesive qualities are exhibited by common polymers, such as poly (acrylic acid), chitosan, and cellulose derivatives, through Van der Waals forces, hydrogen bonds, and electrostatic interactions. To treat dysplastic lesions and prevent further mucosal damage, these materials enhance adhesion and facilitate controlled medication release, thereby reducing the frequency of reapplications and maintaining therapeutic levels. As a localized, non-invasive medication administration technique, mucoadhesive patches offer clear benefits, particularly in the challenging mouth cavities. When treating chronic or recurrent disorders, such as Oral Potentially Malignant Disorders (OPMLs), these patches offer a comfortable and simple administration that improves patient compliance and permits at-home treatment [7].

**Figure 2:**
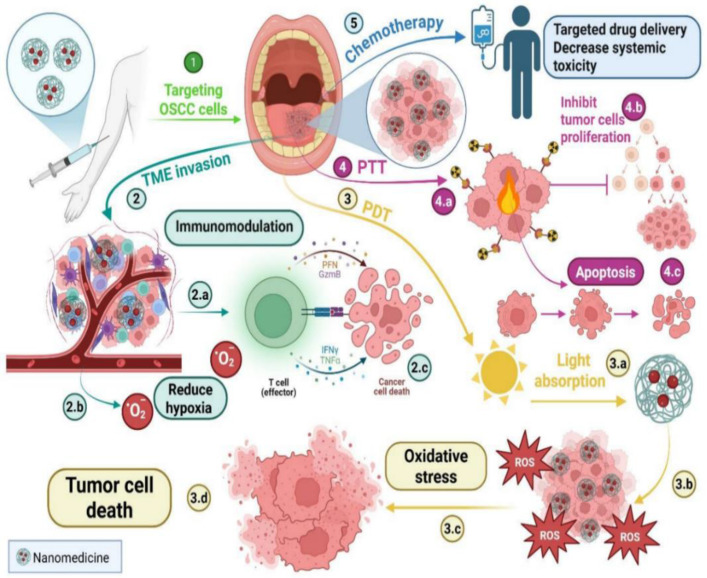
Schematic illustration of nanomedicine-based therapeutic strategies against OSCC. Multiple mechanisms, including TME modulation, photodynamic and photothermal therapy, induction of oxidative stress, apoptosis, and targeted chemotherapy, are used to enhance tumor cell death while reducing systemic toxicity. 2.a: Immunomodulation, 2.b: Reduction in hypoxia by producing oxygen, 2.c: Cancer cell death by the immune system, 3.a: Light absorption by the effect of PDT, 3.b: Production of ROS, 3.c: Oxidative stress inside the tumor cells, 3.d: Tumor cell death due to oxidative stress, 4.a: PTT induces hyperthermia inside the tumor cells. 4.b: Inhibition of tumor cell proliferation by the PTT effect, 4.c: Tumor cell apoptosis by PTT (created by biorender.com).

Size, charge, and ligand decoration influence cellular uptake, or the pathway. A bovine milk exosome (EXO)-based EXO–doxorubicin (DOX)–anthracene endoperoxide derivative (Exo@Dox-EPT1) was developed to significantly enhance cellular uptake, pH- triggered DOX release, and ROS generation upon 808 nm near-infrared laser stimulation, creating a pH/light-sensitive drug system based on milk-EXO for OSCC therapy. Significant control over drug release, biocompatibility, and OSCC cell proliferation was demonstrated by this novel milk-EXO-based drug delivery system (DDS) [28]. Nanomedicine limits the systemic toxicity of chemotherapy treatment, as mentioned previously. In addition, nanomedicine increases the effectiveness of PDT in treating OSCC by improving solubility, stability, and tumor localization; light activation generates ROS, causing apoptosis and necrosis in tumor cells while sparing normal tissues [29], as illustrated in Figure 2.

The drawbacks of traditional PDT, such as inadequate light penetration into deeper tissues and low photosensitizer (PS) solubility, can also be mitigated by these nano-based systems. Nanotechnology enables better control over treatment depth and increased PS accumulation at the tumor site. It has been shown that using NPs, such as upconversion NPs, quantum dots, and gold NPs, enhances ROS production and light absorption [29]. These nanomaterials possess unique optical properties, including surface plasmon resonance and upconversion luminescence, which allow for more efficient tumor ablation and deeper tissue penetration. As a result, improving PDT’s photodynamic effects often yields better treatment outcomes. Through surface plasmon resonance, MONPs further enhance light absorption and ROS generation [29]. In addition to its effects on chemotherapy and PDT, nanomedicine also significantly impacts PTT and immunomodulation. PTT inhibits tumor cell proliferation, but can also cause tumor cell necrosis and degeneration [30].

## 5. Metal Oxide Nanoparticles Used for Oral Cancer

A nanoparticle is a subtype of nanomaterials that displays all three dimensions in the nanoscale size (~1–100 nm) [31]. NPs are utilized in oral cancer treatment because they overcome many limitations associated with conventional therapies, including chemotherapy, radiotherapy, and surgery. NPs can be classified according to various criteria, such as composition (lipid, polymeric, inorganic, carbon-based, etc.), dimensionality, surface properties, application, targeting mechanism, or origin (chemical, physical, or green synthesized).

Understanding the dimensionality of NPs is essential for determining the drug-loading mechanism. NPs occupy all three spatial dimensions (x, y, z) within the nanoscale, enabling visualization of their complete three-dimensional structure [32]. Their dimensions influence drug-loading strategies, either via surface adsorption or encapsulation [32]. The choice between either having the drug adsorbed or encapsulated in an NP depends on several factors, including the physicochemical properties of the drug (such as solubility, molecular size, and stability) and the desired therapeutic release profile [33]. Adsorbed drugs on the NP surface are typically designed for immediate response to environmental triggers (pH or enzymes), high loading efficiency, and maintenance of bioactivity [34], whereas encapsulated drugs provide sustained and controlled release, enhanced bioavailability, minimized side effects, improved delivery, imaging capabilities, and targeted therapeutic effects [35].

NPs can also be classified according to their surface characteristics, which critically affect tissue penetration, cellular uptake, and clearance [36]. Key parameters include: size, which governs tissue penetration and cellular uptake; shape (spheres, rods, discs, core–shell, hollow), which influences circulation, margination, and tumor accumulation; surface charge, which affects interactions with cell membranes and serum proteins, as well as NP stability in suspension [37]; and surface chemistry, as it determines the functional groups present on the surface of NPs (carboxyls, thiols, etc.), which determines the hydrophobicity and hydrophilicity of the NPs; and more [38]. NPs are integrated into various clinical applications, including early and concise diagnosis, enhanced therapeutic efficiency, improved stability, theranostic capability, and improved survival outcomes [39]. Comprehending NP targeting mechanisms is critical for the creation of concise and effective cancer treatment, because they govern how NPs selectively distribute therapeutic chemicals to tumor areas while causing minimal harm to healthy tissues. Targeting strategies can be classified into passive, active, and stimuli-responsive mechanisms, as shown in Table 2.

Several NPs, including iron oxide (FeO), magnesium oxide (MgO), and ZnO, have shown great promise in treatment of oral cancer. These NPs exert cytotoxic effects on cancer cells by including apoptosis, disrupting essential biological processes, and generating ROS. ZnONPs specifically target cancer cells by releasing zinc ions and promoting ROS production. Plant extracts can be employed as reducing and capping agents. ZnONPs have been shown to selectively induce cytotoxicity in cancer cells while sparing healthy cells.

Moreover, these technologies hold promise in diagnostics and imaging, potentially facilitating the early detection of malignant cells [41], as shown in Table 3. MgONPs synthesized using plant extracts also exhibit significant anticancer activity by generating ROS, inducing apoptosis, and enhancing the efficacy of conventional chemotherapeutic agents. The therapeutic outcomes can be further improved through synergistic combinations of MgONPs with standard chemotherapy. Additionally, MgONPs have shown potential in overcoming multidrug resistance in cancer cells, which represents a major obstacle in effective cancer treatment [42].

Nanomaterial synthesis methods can be classified into two types: traditional methods and green methods. Traditional nanomaterial manufacturing methods offer numerous appealing benefits. These technologies yield a wide range of NPs with multiple applications [48]. Some technologies provide significant scalability and precise control over NP morphology, with applications in novel battery conduction, electrical applications, targeted disease therapy, and energy storage/conservation. However, the adverse impacts of these traditional approaches are clear. Organic solvents are extensively utilized in synthesizing these nanomaterials, which pose a significant neurobehavioral and reproductive risk during the process. Additionally, the use of high-pressure and heat conditions may contribute to hazardous working conditions. One of these syntheses’ most important adverse effects is the concern for volatile vapor and excessive carbon dioxide production, which significantly contributes to the greenhouse effect. All things considered, these techniques endanger the environment and the scientists performing the synthesis in irreparable ways. These possible drawbacks outweigh the advantages of conventional nanomaterial manufacturing techniques. Green synthesis has become more popular because of the decline in popularity of traditional synthesis techniques.

**Figure 3:**
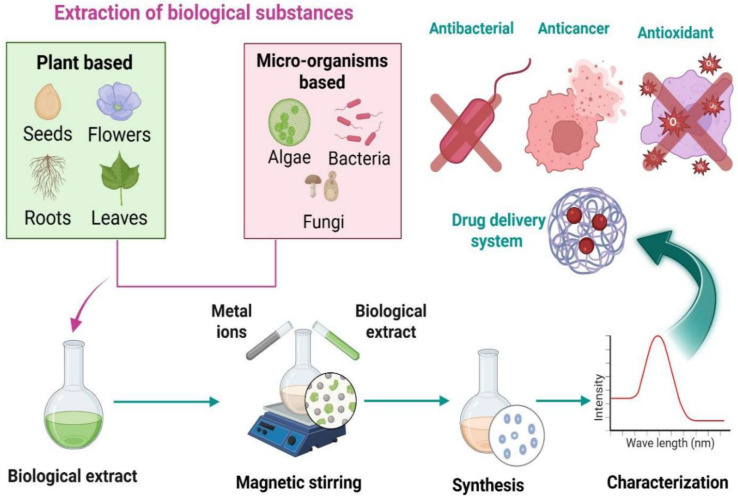
Green synthesis of NPs by using biological sources (created by biorender.com).

Green synthesis is an environmentally friendly, economical, safe, and clean method of creating nanomaterials. The green synthesis of nanomaterials uses microorganisms as substrates, including bacteria, yeast, fungi, algae, and some plants, as illustrated in Figure 3. Several active molecules and precursors, including metal salts, determine the ultimate shape and size of the NP. The advantages of green synthesis for nanomaterials include inherent reducing, stabilizing, and antibacterial qualities. There are no harmful impacts on the environment, and the biological process is considered safe and economical [49].

In contrast, using harmful compounds and creating toxic byproducts in chemical and physical methods has several negative environmental repercussions. Among the many benefits of biosynthesized NPs are their excellent stability and reduced toxicity. Furthermore, the biosynthesized NPs have a broad spectrum of medicinal uses, such as antibacterial, anti-inflammatory, anti-cancer, and antioxidant agents, as well as prospective carriers for different drug delivery systems [50].

Several variables influence the process of creating NPs from plant extracts. For instance, it is necessary to optimize the concentration of plant extract. The right amount of plant extract improves the size, shape, and synthesis of NPs. The reaction temperature is another essential component that directly impacts the size and structure of NPs. Furthermore, the reaction temperature has a direct impact on the rate of reaction, which in turn influences the properties of the NPs. Therefore, changing the reaction’s temperature may modify the desired characteristics, such as size, shape, growth, and particle distribution. Furthermore, the size and shape of NP production are all influenced by the pH of the fluid. As the pH rises, more nucleation centers are created, which causes the metal ions to change into their solid metallic state. The pH of the solution influences the activity of the functional groups in the plant extract, which speeds up the reaction rate. The ability of plants to produce MONPs from their inorganic metal ions has been the subject of much recent research. The reduction in metal ions also depends on the phytochemicals present in plants. Utilizing plant extracts in manufacturing MONPs has several advantages, including lower maintenance and waste disposal expenses, less creation of toxic waste, favorable effects on treatments, and the extracts’ dual functions as stabilizing and reducing agents [51].

Green-synthesized MONPs often show comparable or superior quantitative performance in oral cancer treatment compared to conventionally synthesized ones, primarily due to their smaller size, different morphology, and inherent biocompatibility. Specific examples include green-synthesized Ag_2_O particles using *Datura innoxia* leaf extract, exhibiting higher anticancer activity. Green-synthesized Ag_2_O particles showed significantly higher anticancer activity against cancer cells, with a lower IC_50_ value of 17.908 µg/mL compared to 23.856 µg/mL for chemically synthesized particles [52]. ZnO-NPs are synthesized utilizing bud extract (CBE) from *Syzygium aromaticum* (clove). At lower doses, CBE-ZnO-NPs showed a little decrease in tumor cell metabolic activity; at higher concentrations, the decline was more noticeable. On HNO-97 cells, the IC_50_ value for CBE-ZnO-NPs was found to be 73.35 µg/mL. The presence of bioactive chemicals from the clove extract adsorbed onto the NP surface, which increases their therapeutic potential, probably causes the anticancer action of CBE-ZnO-NPs [53]. In the culture supernatant, bacteria release various bioactive substances crucial to NP formation, such as enzymes, proteins, hormones, ions, polysaccharides, and pigments. Sulfur-containing proteins and nicotinamide adenine dinucleotide (NADH)-dependent reductases are crucial for the stability and reduction in NPs [50]. 

Because of their ability to reduce metal ions, the production of NPs from a variety of algal resources has emerged as one of the most cutting-edge and current fields of biochemical study. The algae species and mode of operation determine whether intracellular or extracellular synthesis is used to create the NPs. The newest and most promising species employed in creating nanomaterials are algae, particularly microalgae. Compared to other living things or biomaterials, algae are a more promising choice for synthesizing nanomaterials. To develop diverse algae species, the researchers employed various techniques, including closed cultivation systems as photobioreactors and open culture systems as raceway ponds, tanks, and open ponds. In most experiments, the following primary processes are observed in the production of MONPs utilizing algae: boiling or heating an algal extract for a predetermined amount of time in water or an organic solution; making molar solutions of ionic metallic compounds, and under controlled conditions, algae and ionic metallic compound solutions are incubated for a specific amount of time, either with or without frequent stirring [54].

Nanomaterials were characterized by using a variety of characterization tools, including atomic field microscopy (AFM), scanning electron microscopy (SEM), field emission scanning electron microscopy (FESEM), transmission electron microscopy (TEM), high-resolution transmission electron microscopy (HRTEM), X-ray diffraction (XRD), energy dispersive X-ray analysis (EDAX), particle size distribution (PSD), zeta potential (ZP), photoluminescence (PL), dynamic light scattering (DLS), raman spectroscopy (R), infrared spectroscopy (FTIR), cyclic voltammetry (CV), nanoparticle tracking analysis (NTA), thermal gravimetric analysis (TGA), and electron diffraction (SAED) [55], as shown in Table 4.

## 6. Delivery Systems Based on Green Synthesized Metal Oxide Nanoparticles

Cancer treatment is complex because of issues like cytotoxicity to healthy, normal cells, medication resistance, and a continual lack of selectivity. A potent instrument for improving cancer diagnosis and treatment is nanotechnology. DDs are designed to safely transport therapeutic drugs within the body to achieve the intended therapeutic effect, as shown in Table 5. These systems are typically developed to enhance active compounds’ aqueous solubility and chemical stability, boost pharmacological activity, and minimize side effects—NPs as carriers have shown significant promise in recent years. Drug encapsulation in NPs, such as micelles, liposomes, dendrimers, nanocapsules, and nanospheres, enhances the therapeutic index and decreases adverse effects. For instance, liposomal DDS can improve bioavailability, increase effectiveness, and lower toxicity [69].

DDs increase therapy versatility because they can be given through various routes, including oral, nasal, parenteral, and intravenous. The ideal NP size and surface characteristics of DDSs extend circulation duration and allow for regulated, sustained drug release at target areas and during transit, improving the biodistribution of cancer medications. Additionally, DDSs address issues with multidrug resistance, poor selectivity, and low water solubility that arise in traditional cancer therapies by increasing intracellular drug concentration through processes including EPR or endocytosis. Targeted therapy seeks to minimize side effects and collateral damage to rapidly dividing normal cells by delivering chemotherapeutics specifically to cancer cells. This accuracy addresses methods’ drawbacks while improving cancer treatments’ effectiveness [3].

MONPs enhance biodistribution and pharmacokinetics in a variety of ways. By reducing opsonization by the reticuloendothelial system (RES) and extending circulation time, surface functionalization using biocompatible polymers like PEG [70], dextran [71], or PLGA [72] produces a “stealth effect,” improving biodistribution and retention in target tissues. Targeting with an external magnetic field makes delivery more precise. By improving drug stability, enabling targeted transport to cancer cells, and facilitating regulated release, MONPs are crucial for enhancing the release and efficacy of anticancer drugs [73]. These carriers serve as vehicles to encapsulate and release anticancer medications in a controlled manner; they frequently lack intrinsic therapeutic qualities [74].

The main advantage of using MONPs is that they protect the encapsulated anticancer medication from rapid metabolism, breakdown, and excretion by the body, which increases bioavailability and reduces systemic toxicity [3]. MONPs exhibit significant cytotoxic potential against oral cancer cells by generating ROS that induce apoptosis and suppress tumor progression. Their excellent stability, biocompatibility, and modifiable surface properties make them highly suitable for targeted drug delivery and advanced diagnostic applications [1].

The subsequent section presents a comprehensive Table 5 summarizing studies on green-synthesis of MONPs used for the delivery of anticancer drugs for various cancers. No prior research has focused explicitly on their application in oral cancer therapy, underscoring a critical gap in current knowledge. This unexplored area represents a promising avenue for future investigation, with the potential to introduce sustainable, efficient, and targeted treatment strategies for oral cancer management.

**Table 5:** Delivery system based on green-synthesized nanomaterials for cancer treatment.

Nanoparticle	Biological Source	Drug	Model	Ref.
CuO-ZnONPs	Trichosanthes dioica fruit extract	5-fluorouracil	HeLa cells	[75]
CuONPs	Trichosanthes dioica dried seeds extract	5-fluorouracil	HeLa cervical cancer cells	[76]
ZnONPs	Microwave-Assisted and Gambogic Acid-Mediated Processes	Asplatin	MDA-MB-231 breast cancer cells	[77]
Fe_3_O_4_@PEG	Musa paradisiaca peel extract	Doxorubicin	HeLa tumor line	[78]
ZnONPs L1, L2, L5, L10	Azadirachta indica leaf extract	Cisplatin	Cervical squamous carcinoma cell line SiHa and murine macrophage cell line RAW 264.7	[79]
ZnONPs	Fungus Aspergillus niger	Doxorubicin	A549 cells	[80]
ZnONP	Ethanolic extract of *Camellia sinensis* L.	Paclitaxel	MCF-7 cell line	[81]
Fe_3_O_4_NPs	Guava leaves extract	Doxorubicin	Human red blood cells	[82]
ZnONPs	Borassus flabellifer fruit	Doxorubicin	MCF-7 cells	[83]

## 7. Overcoming the Limitations of Herbs Used for Oral Cancer Treatment

Herbal products are now commonly available in the market under different regulatory categories. They are increasingly preferred in primary healthcare over conventional medicine due to fewer side effects and better tolerance. However, delivering herbal ingredients remains a challenge [84]. For example, essential oils are volatile, limiting their use. In addition, topical application may cause irritation or sensitivity to the oral mucosa, restricting their usage. Moreover, other issues with herbal therapies, including poor solubility, low permeability, prolonged treatment duration, low bioavailability, and additional challenges, reduce their effectiveness. Medicinal plants contain multiple potential components for treating oral-dental diseases. Because of their long history of effectiveness, people often use herbal ingredients without caution. It is generally assumed that herbal remedies are safer than allopathic medicine. However, the belief that herbal products are free from toxic side effects is not always accurate.

Allergic reactions to essential oils must be considered. Research indicates that essential oils from sandalwood, lavender, tea tree, and clove are most likely to cause irritation and inflammation. The main components responsible include benzyl alcohol, geraniol, eugenol, and hydroxyl-citronella. High concentrations or doses of essential oils may lead to adverse reactions [85]. Factors such as the biological content, source of material, and route of exposure should also be evaluated for irritation risk. Recent studies show that herbal extracts can cause adverse effects even at low doses. Though recognized for their medicinal value and currently used in treatments, some plants display toxic effects. In addition, synergistic use of herbal ingredients for improved therapeutic results may increase the overall concentration of chemical constituents, leading to higher toxicity risks. Nonetheless, the presence of side effects does not mean herbal medicines should be avoided. Safe use can be achieved through pharmacological screening and proper evaluation of preparation components.

Despite the proven therapeutic efficacy of herbal products, patient acceptance remains an essential factor. Since these products are intended for dental disease treatment, good taste, smell, and other organoleptic properties are necessary. Essential oils cannot be taken orally and are limited to local applications such as gargles, mouthwashes, and ointments. Their main drawback is the strong odor. Tea tree oil (*Melaleuca alternifolia*) demonstrated antimicrobial activity in 34 patients, but when its organoleptic properties were compared with Colgate toothpaste, it produced an unpleasant taste. Likewise, tea tree oil-based mouthwashes showed poor taste and a stinging sensation in the mouth. Although most essential oils are used to mask oral disease-related smells due to their strong odor, they are still often rejected by consumers. Eucalyptus and tea tree oil, known for their strong odor, are especially poorly accepted [85].

In developing herbal drug products, achieving permeability of drug molecules across the epithelial mucosal barrier is necessary for effective therapeutic action. Differences in permeability occur depending on the oral mucosa site. Keratinized regions contain ceramides that block hydrophilic drugs, while non-keratinized areas restrict hydrophobic drug permeation. Saliva flow in the mouth also acts as a barrier to proper delivery. Additionally, the instability of herbal active compounds in the gastric region is a concern. Most isolated herbal constituents are hydrophobic and poorly soluble, resulting in low bioavailability, which must be considered for effective action [86]. This often requires higher doses, leading to adverse effects and poor patient compliance. Phenolic-based plant constituents, being water-soluble, have limited absorption across lipid membranes.

Furthermore, unsuitable molecular size contributes to poor absorption. Chinese medicines, which often contain large molecules, face difficulties in absorption, affecting other physicochemical properties. Though small enough to permeate and absorb, essential oils undergo rapid metabolism and have short half-lives, leading to low bioavailability. Marketed compounds like curcumin and ellagic acid show poor bioavailability due to low solubility in aqueous media and extensive metabolism. In a rat study, oral administration of 400 mg curcumin showed no detection in plasma, with only trace amounts in portal blood [87]. Therefore, while many plant-based drugs demonstrate strong potential in vitro, they often fail to perform in clinical stages due to poor bioavailability.

A significant limitation of most herbal medicinal products is their short duration of action. Formulation scientists must consider minimizing the dosing frequency of these products. Efforts are ongoing to enhance both the duration and onset of action of herbal medicines [88]. Herbal products are sold under different categories across the world. Several regulatory classifications exist, including over-the-counter drugs, prescription drugs, traditional medicines, and dietary supplements. There is a need for strict global and regional regulatory frameworks to monitor herbal medicinal products. The extent of quality, safety, and efficacy data required for registration differs by region, highlighting the need for harmonized requirements. Many herbal products on the market still lack proven evidence of safety and efficacy. Inadequate cultivation, harvesting, and storage methods further stress the importance of standardizing herbal preparations. Another primary concern is contamination with heavy metals during cultivation and adulteration of herbal ingredients. World Health Organization (WHO) has taken the lead in defining parameters for herbal medicinal products’ quality, safety, and efficacy to establish evaluation standards. Basic evaluation parameters for herbal drug products have also been incorporated into pharmacopeial monographs. Ongoing research focuses on developing herbal medicines with maximum bioavailability and effective concentration at target cells.

Thus, an appropriate delivery system is essential to realize the potential of natural products [88]. The therapeutic potential of natural compounds is often restricted due to their poor water solubility, limited bioavailability, and inadequate targeting ability [89]. The significant drawbacks of curcumin are its low water solubility and poor bioavailability [90]. Curcumin challenges due to poor solubility in aqueous environments, susceptibility to hydrolysis, and consequently low bioavailability [91]. Curcumin exhibits low systemic absorption and undergoes rapid metabolic breakdown, resulting in restricted therapeutic outcomes [91]. The therapeutic application of curcumin is limited by its poor water solubility and low bioavailability, resulting from rapid metabolism and clearance [92]. Curcumin, a well-known phytochemical extracted from Curcuma longa, possesses broad-spectrum anticancer properties. Nevertheless, its therapeutic potential is hindered by poor bioactivity, limited solubility, and low chemical stability, leading to insufficient cellular uptake in cancer cells [93].

Few studies have been conducted to explore the link between green tea and the prevention or treatment of oral cancer. A case–control study indicated that individuals who consumed one or more cups of green tea daily had a 37% lower risk of developing oral cancer than those who did not drink tea. However, more significant results were observed among individuals who consumed five or more cups of green tea daily compared to those who drank less than one cup daily. A 2013 study reported that patients who regularly consumed green tea extracts in supplement form experienced a suppression of the adverse effects of malignant tumors. In one study involving 59 patients who were given 3 g of a mixed green tea extract daily, around 37.9% showed a decrease in wound size. In contrast, only 3.4% experienced an increase in tumor size, suggesting an inverse relationship between green tea polyphenols and the risk of oral cancer [94].

It is well established that reducing the particle size of herbal bioactives improves solubility and dissolution. According to the target site of action, formulation size can be adjusted to support transport across the biomembrane. As the bioavailability of poorly soluble drugs is limited by dissolution, even a slight increase in solubility greatly influences bioavailability. For example, although it has vigorous anti-inflammatory activity, curcumin shows poor oral absorption in powder or conventional delivery forms due to its hydrophobic nature. To address this, nanomicelles were developed to encapsulate curcumin in a hydrophobic core, making it water miscible. The application of nanotechnology to improve therapeutic performance is well recognized. Studies have shown that nanosizing formulations increases the permeation and bioavailability of phytoconstituents. For example, microspheres of zedoary oil from turmeric were developed, where the small particle size improved in vivo absorption and raised bioavailability. Additionally, sustained release minimized adverse effects and reduced dosing frequency. Nanotechnology has also been effective in enhancing the stability of essential oils by protecting them against oxidation, hydrolysis, photodegradation, thermal degradation, and reducing volatility. Since low aqueous solubility and high volatility restrict the direct use of essential oils, encapsulation into delivery systems becomes necessary [88].

The integration of herbal medicine with conventional therapeutic agents has gained increasing attention in recent years, particularly when combined with nanomaterials as delivery platforms, as shown in Table 6. Such combinations aim to enhance drug solubility, stability, and bioavailability while exploiting herbal extracts’ synergistic pharmacological effects. By loading traditional drugs and phytochemicals onto NPs, this innovative strategy enhances therapeutic efficacy against oral cancer. It holds promise for developing more effective treatment approaches, ultimately leading to better patient outcomes [92].

**Table 6:** Loading herbs with a traditional drug.

Nanoparticle	Characterization Technique	Drug + Herb	Model	Advantages	Ref.
Niosome NPs	DLS, ZP, Atomic Absorption, F-4500 fluorescence, and HPLC	Cisplatin + Curcumin	CAL27 Cell	- Improve solubility and bioavailability. - Minimize adverse effects. - Ability to bypass or reduce drug resistance. - Controlled, long-lasting drug release. - Enhanced stability. - Precise and targeted delivery.	[92]
PLGA NPs	TEM and DLS	Cisplatin + Curcumin	CAL27, CAR cells, HGFs, and OKs	- Promote apoptosis. - Minimal toxicity toward normal cells. - Enhances the solubility in water and bioavailability.	[90]
Nanoemulsion	ZP, PDI, TEM, UV-vis, EE and DLS	5-Fluorouracil + Curcumin	SCC090 and SCC152	- Improved bioavailability. - Provides a controlled and sustained release profile. - Ensures higher intracellular uptake. - Strongly induces apoptosis. - Displays safety.	[91]
Nanoliposomes	ZP and PDI	Carboplatin and Cisplatin + Curcumin	CAL 27 oral cancer cells	- Improve cytotoxic effects. - Stable, sustain.	[95]
Phytosomes	TEM, FTIR, DSC, Solubility study, Partition coefficient (Po/w), and Colloidal properties assessment	Cisplatin + Tetrahydrocurcumin	SCC4 cell line, Normal oral keratinocytes, and Gingival fibroblasts	- Improve aqueous solubility with favorable colloidal stability. - Complete drug release within one hour. - Stronger cytotoxic activity and greater selectivity toward cancer cells. - Exhibited potent free radical scavenging activity.	[93]

## 8. Conclusions

Effective NP delivery in OSCC remains hindered by tumor heterogeneity and the unreliable EPR effect, necessitating a shift toward mechanism-driven, patient-adapted strategies. Although green synthesis of MONPs offers sustainability and cost advantages, batch variability, limited reproducibility, and unclear biodegradation hinder clinical translation. Standardized synthesis protocols and long-term toxicological assessments are urgently required. Rational surface engineering, including ligand conjugation and polymeric coatings, can improve immune evasion, targeting precision, and systemic retention. Integrating omics-based biomarker profiling with scalable green nanomanufacturing may balance personalization with practicality. To achieve regulatory and clinical acceptance, harmonized standards, GMP-compatible production, and advanced evaluation models—such as tumor-on-chip and computational toxicology—should be implemented. Through interdisciplinary collaboration and continuous refinement, eco-synthesized MONPs hold strong potential as sustainable and clinically viable nanotherapeutics for oral cancer management.

## Figures and Tables

**Table 1:** Advantages and disadvantages of some traditional drugs used in oral cancer therapy.

Traditional Drug	Advantages	Disadvantages	Mechanism	Ref.
Cisplatin	- Reduced tumor volume and improved overall prognosis.	- Nephrotoxicity. - Common side effects include anaphylaxis, cytopenias (including leukopenia and neutropenia, thrombocytopenia, and anemia), hepatotoxicity, ototoxicity, cardiotoxicity, nausea and vomiting, diarrhea, mucositis, stomatitis, pain, alopecia, anorexia, cachexia, and asthenia.	Generation of DNA lesions followed by the activation of the DNA damage response and the induction of mitochondrial apoptosis.	[8,9,10]
“Taxanes” (Paclitaxel and Docetaxel	- Function as antimitotics but also impair diverse oncogenic signaling pathways, including angiogenesis, inflammatory response, ROS production, and apoptosis induction.	- Extreme hydrophobicity, low water solubility, low bioavailability, and high toxicity.	Work by disrupting microtubule assembling dynamics and inducing cell cycle arrest at the G2/M phase of the cell cycle, ultimately triggering apoptosis.	[11,12,13]
5-fluorouracil	- Inhibits cancer growth. - Enhanced efficacy with sequential treatment.	- Common resistance limits clinical effectiveness.	Inhibiting thymidylate synthase also serves as a pyrimidine analog by misincorporating into RNA and DNA instead of uracil or thymine.	[14,15]
Methotrexate	- Reduction in tumor size of more than 50%. - Reduced need for reconstructive surgery. - Decrease recurrence rate. - Enhanced operability and morbidity.	- Short-term follow-up. - Potential variability in response.	Promotion of adenosine release and inhibition of transmethylation reactions.	[16,17]
Carboplatin	- Well-tolerated with manageable side effects. - Converted many inoperable oral cancers into operable ones. - Enhanced survival rates. - Feasible and practical with limited medical resources.	- Potential toxicities, both hematologic and non-hematologic, need to be monitored.	It contains the metal platinum, which binds to and damages the cell’s DNA, preventing it from being repaired or copied. This stops cells from dividing and causes them to die.	[18]
Bleomycin	- Local tumor control. - Enables reasonable response rates. - Reduced systemic toxicity, as the required drug dose can be lowered due to its enhancement effect.	- Pulmonary fibrosis. - In the context of electroporation, there may be some anatomical and technical limitations. - Limited evidence base in oral cancers. - Systemic use may induce dose-limiting toxicities.	Binds to guanosine-cytosine-rich portions of DNA via association of the “S” tripeptide and by partial intercalation of the bithiazole rings. A group of five nitrogen atoms arranged in a square pyramidal conformation binds divalent metals including iron, the active ligand, and copper, an inactive ligand. Molecular oxygen, bound by the iron, can produce highly reactive free radicals and Fe (III). The free radicals produce DNA single-strand breaks at 3′-4′ bonds in deoxyribose. This yields free base propenals, especially those of thymine; cytotoxicity is cell-cycle-phase specific, with the G2 phase being the most sensitive.	[19]
Pembrolizumab	- Improves overall survival. - Meaningful objective responses. - Good tolerability in a surgical setting. - Enables immune activation and tumor micro-environment change, enhancing the potential for a more profound effect.	- Benefit depends on biomarker (PD-L1) status. - Some patients may not respond to treatment even in small series, as some patients progressed early. - May cause immune-mediated toxicities. - Expensive and may not be readily available in all settings, which can limit its applicability in resource-constrained environments.	It binds to the protein PD-1 on the surface of specific immune cells called T cells, which keeps cancer cells from suppressing the immune system. This allows the immune system to attack the cancer cells.	[20]
Erlotinib + celecoxib + methotrexate	- The patients studied achieved a 3-month progression-free survival rate of ~71.1%. - The response rate (partial + complete) was ~42.9%. - Improvements in quality of life.	- The median follow-up was relatively short (~6.8 months), limiting knowledge about long-term durability. - The regimen was studied in a single-arm, non-randomized trial, so there is no direct comparator arm. - Some toxicities and adverse events led to treatment discontinuation. - In longer-term follow-up, results were less favorable.	Erlotinib works by blocking the action of a protein called EGFR that signals tumor cells to multiply. This helps slow or stop the spread of tumor cells. Celecoxib may specific the growth of tumor cells by blocking certain enzymes required for cell growth. Methotrexate prevents cells from utilizing folic acid to synthesize deoxyribonucleic acid, potentially killing tumor cells.	[21]
Doxorubicin	- Highly potent anticancer agent. - Targets rapidly dividing cells. - Enhance therapeutic efficiency when encapsulated. - Reduce toxicity with nanocarriers.	- Lack of selectivity. - High toxicity. - Clinical limitation.	Bind to DNA-associated enzymes, intercalate with DNA base pairs, and target multiple molecular targets to produce a range of cytotoxic effects. For instance, it activates various molecular signals from AMPK (AMP-activated protein kinase) to influence the Bcl-2/Bax apoptosis pathway, inducing apoptosis. By altering the Bcl-2/Bax ratio, downstream activation of different caspases can occur, resulting in apoptosis.	[22,23]

**Table 2:** Comparative overview of targeting strategies in oral cancer.

Targeting Type	Mechanism	Advantages	Disadvantages	Evaluation	Ref.
Passive Targeting	- EPR effect in tumor vasculature and poor lymphatic drainage.	- Non-complex formulations. - Useful for initial drug accumulation in oral cancer.	- Ineffective in poorly vascularized or hypoxic oral cancer tumors. - Rapid clearance by RES.	Insufficient for precise oral cancer treatment due to their extensive vasculature and tumor microenvironment	[28]
Active Targeting	- Uses antibodies, peptides, folate, or hyaluronic acid to bind overexpressed receptors on oral cancer cells (EGFR, CD44, folate receptors).	- Increases tumor cell specificity and uptake. - Reduction in systemic toxicity.	- Premature drug release. - Requires validated tumor biomarkers, as it may limit the universal application.	Lacks control over release but is more effective than passive Targeting and depends on biomarker selection.	[28]
Stimuli-responsive Targeting	- Releases drug in response to internal triggers of the tumor (Ph, ROS, enzymes) and external triggers (temperature, light). - Enabling controlled release in oral cancer.	- Site-specific release, which minimizes systemic toxicity. - Enhance combination therapies.	- Not fully cell-specific if used alone, requires external stimulus delivery.	Improves precision, especially when combined with active targeting.	[40]

**Table 3:** Anticancer and targeted drug delivery of green-synthesized MONPs against oral cancer cell lines.

Nanoparticle	Biological Source	Model	Efficiency	Ref.
ZnONPs	Curcumin	Human Oral Epidermal Carcinoma KB cells	Significantly upregulate key apoptotic genes such as BCL2, BAX, and P53.	[43]
ZnONPs	Piperine	KB oral squamous carcinoma cells	Significant upregulation of essential apoptotic genes, which elucidates the complex mechanism by which nanoparticles induce apoptosis.	[44]
ZnONPs	κ-carrageenan	Human Oral Epidermal Carcinoma	Induced apoptosis in KB cells involved an increase in the expression of the BCL-2, BAX, and P53 genes, which induced apoptosis in KB cells.	[45]
ZnONPs	Cinnamomum verum bark extract	Oral KB cells	CV-ZnONPs induced apoptosis in KB cells by reducing viability, increasing ROS levels, and enhancing caspase activity.	[46]
ZnO-Ag nanocomposite	Stenotaphrum secundatum grass extract	Oral squamous cell carcinoma (CAL 27) cells	ZnO-Ag NCs exhibited potent anticancer activity against CAL 27 cells (IC_50_ value: 15 ± 1.1 µg/mL), inducing apoptosis and G2/M cell cycle arrest, which highlights their potential for cancer and biomedical applications.	[47]

**Table 4:** Characterization techniques for NPs.

Techniques	Purpose	Ref.
UV-Vis	Optical properties, size, concentration, and agglomeration state hint at NP shape.	[56]
XRD	Crystal structure, composition, and crystalline grain size.	[56]
FTIR	Surface composition, and ligand binding.	[56]
R	Analyze chemical composition and nanoscale biological interactions.	[57]
FESEM	Capture the microstructure image of the materials.	[58]
DLS	Hydrodynamic size and detection of agglomerates.	[56]
ZP	Used to assess NP surface charge and colloidal stability, which is essential for accurate characterization and successful drug-delivery formulations.	[59]
SEM-EDAX	Morphology, dispersion of NPs in cells and other matrices/supports, precision in lateral dimensions of NPs, and quick examination–elemental composition.	[60]
TEM-HRTEM	NP size, size monodispersity, shape, aggregation state, detect and localize/NPs in matrices, and study growth kinetics.	[61]
AFM	NP size and shape in 3D mode, evaluate the degree of covering of a surface with NP morphology, dispersion of NPs in cells and other matrices/supports, precision in lateral dimensions of NPs, and quick examination–elemental composition.	[62,63]
PL	Study the chemical reaction of molecules on solid surfaces and the reactivity of various heterogeneous solid catalysts in relation to their properties in adsorption, catalysis, and photocatalysis.	[64]
NTA	NP size and size distribution.	[65]
TGA	Mass and composition of stabilizers.	[65]
PSD	Measure the diffusion coefficient of particles as small as 30 nm in diameter, characterize changes in particle size and distribution as a function of small, label-free, and surface modifications of particles.	[66]
SAED	Study the structure of materials.	[67]
CV	Investigate the reduction and oxidation processes of molecular species.	[68]

## Data Availability

No new data were created or analyzed in this study.

## Abbreviations

OSCC	Oral Squamous Cell Carcinoma
ROS	Reactive Oxygen Species
EPR	Enhanced Permeability and Retention effect
TME	Tumor Microenvironment
EXO	Exosome
DOX	Doxorubicin
DDS	Drug Delivery System
PDT	Photodynamic Therapy
PS	Photosensitizer
PTT	Photothermal Therapy
RES	Reticuloendothelial System
NADH	Nicotinamide Adenine Dinucleotide—Reduced Form
AFM	Atomic Force Microscopy
SEM	Scanning Electron Microscopy
FESEM	Field Emission Scanning Electron Microscopy
TEM	Transmission Electron Microscopy
HRTEM	High-Resolution Transmission Electron Microscopy
XRD	X-ray Diffraction
EDAX	Energy Dispersive X-ray Analysis
PSD	Particle Size Distribution
ZP	Zeta Potential
PL	Photoluminescence
DLS	Dynamic Light Scattering
FTIR	Fourier Transform Infrared Spectroscopy
CV	Cyclic Voltammetry
TGA	Thermal Gravimetric Analysis
SAED	Selected Area Electron Diffraction
PEG	Polyethylene Glycol
WHO	World Health Organization
MONP	Metal Oxide Nanoparticle
PLGA	Poly lactic-co-glycolic acid
NPs	Nanoparticles
ZnO	Zinc oxide
TiO_2_	Titanium oxide
Fe_2_O_3_/Fe_3_O_4_/FeO	Iron oxide
CuO/Cu_2_O	Copper oxide
OPMLs	Oral Potential Malignant Disorder
CeO_2_	Cerium oxide
R	Raman spectroscopy
MgO	Magnesium oxide
